# Variations in adolescents’ motivational characteristics across gender and physical activity patterns: A latent class analysis approach

**DOI:** 10.1186/s12889-017-4677-x

**Published:** 2017-08-17

**Authors:** Margaret Lawler, Caroline Heary, Elizabeth Nixon

**Affiliations:** 1 School of Psychology, Trinity College, Dublin 2, Ireland; 2 School of Psychology, National University of Ireland, Galway, Ireland

**Keywords:** Adolescent, physical activity, self-determination, motivation, team sport, latent class

## Abstract

**Background:**

Neglecting to take account of the underlying context or type of physical activity (PA) that underpins overall involvement has resulted in a limited understanding of adolescents’ PA participation. The purpose of the present research was to identify male and female adolescents’ leisure time PA patterns and examine whether psychological processes derived from self-determination theory differ as a function of the pattern of PA undertaken.

**Methods:**

Nine hundred ninety-five students (61.2% females, 38.8% males; *M* age = 13.72 years, *SD* = 1.25) from eight secondary schools in Dublin, Ireland completed a physical activity recall 7 day diary and measures of intrinsic motivation, competence, relatedness, autonomy and autonomy support. Based on the diary five binary indicators of physical activity were derived reflecting recommended levels of MVPA on a minimum of 3 days, at least three sessions of non-organized physical activity (e.g. jog), team sport, individual sport, and organized non-sport physical activity (e.g. dance). Latent class analysis was used to identify subgroups of adolescents that engaged in similar patterns of physical activity. Profiles of physical activity participation were subsequently compared on motivational characteristics using Kruskal-Wallis tests.

**Results:**

Latent class analysis revealed six distinct classes for girls (Organized Run/Swim & Dance/Gym; Organized Dance; Leisure Active Team Sport; Active Individual Sport; Walk/Run/Outdoor games; Non-Participation) and five for boys (Leisure Active Gym; Leisure Active Individual Sport; Active Team Sport; Active Mixed Type; Non-Participation). Significant differences were found between the classes. Girls characterized by participation in team or individual sport, and boys represented by team sport participation demonstrated significantly higher self-determined motivational characteristics relative to other profiles of physical activity.

**Conclusion:**

This research offers a nuanced insight into the underlying type of activities that constitute overall patterns of PA among adolescent boys and girls and further reveals that psychological processes vary dependent on the profile of physical activity undertaken. The findings may be useful for informing interventions aimed at promoting physical activity among young people.

## Background

Physical activity (PA) guidelines recommend that young people accumulate at least 60 min of moderate-to-vigorous physical activity (MVPA) daily. Adherence to PA guidelines is associated with a range of positive outcomes including decreased risk of excess weight, cardiovascular disease and psychological difficulties [1–3]. Despite these benefits, most adolescents are insufficiently active [4, 5]. PA is a complex behavior characterized by participation in a number of different types of activity such as sport, exercise and free play. However, previous studies have typically used continuous measures of MVPA or cut-off points to categorize participants into discrete groups reflecting whether or not recommended PA guidelines are met. Focusing on overall PA levels without taking account of the underlying context or type of PA (e.g. team or individual sports; organized i.e. that guided by a coach or instructor, or non-organized activity, such as walking, jogging, informal games) does not allow for examination of groups that differ qualitatively (e.g. active team sport vs active dance), which has further resulted in a limited understanding of the patterns of adolescents’ PA.

Efforts within the literature to investigate underlying types of PA involvement have focused on discriminating the context of participation, which is broadly defined in terms of organized (e.g. team sport, individual sport, PA lessons e.g. dance) and non-organized PA behavior (e.g. informal games of basketball, tag, or exercise such as jogging) [6, 7]. A lack of research exists regarding the relative extent to which non-organized PA contributes to overall PA levels. One longitudinal investigation into male and female adolescents’ PA throughout secondary school revealed that participation in light-to-moderate PA like walking, running, and jogging were more likely to be initiated and continued over time [8], underscoring the potential benefit of this type of PA for promoting adherence to an active lifestyle long-term.

Organized sport in contrast, has been widely researched with empirical studies indicating that participation is associated with higher levels of MVPA among young people [9, 10]. Focusing exclusively on organized sport however, may have precluded an investigation into the role of other organized non-sport PA lessons such as dance or fitness classes (e.g. aerobics, circuits), which may also contribute to total PA levels among youth. In accordance, O’Neill, Pate and Hooker [11] found that female adolescents’ objectively measured activity in structured dance lessons accounted for roughly 29% of their weekly MPVA. Failing to consider organized PA participation alongside sport may therefore result in an underestimation of overall youth PA levels whilst also overlooking an important source of MVPA among girls in particular. As such, it is evident that taking account of the different contexts of PA that underpin overall involvement can provide a greater understanding of adolescents’ PA behavior. Recent studies have attempted to address this issue by adopting person-oriented approaches, which seek to identify groups of adolescents that engage in a similar *pattern* of PA behavior or respond similarly on a set of observed variables, which in turn differentiates them from other subgroups [12]. Identification of different profiles of adolescents’ PA participation may be used to tailor interventions towards specific subgroups of young people promoting increased MVPA. Agans and Geldhof [13] identified five distinct clusters of organized sport participation among a sample of secondary school students that reflected participation in team sport, individual sport, a combination of team and individual sports, dance, and non-participation. However, this study failed to consider involvement in organized non-sport (e.g. gym, aerobics) and non-organized PA (e.g. walking, jogging) yielding an incomplete picture of adolescents’ PA behaviors.

Liu and colleagues [14] identified five distinct groupings of MVPA participation for male and female adolescents as part of a national US Survey. The largest female class, comprising three-quarters of the sample, was labelled ‘dancers/walkers/runners’. In contrast, for males, just less than three-quarters of the overall sample comprised the ‘basketball and runner’ class. The results indicate that preferred type of PA participation differed between boys and girls, highlighting the need to explore gender specific profiles of PA. These findings contribute to our understanding of adolescents’ PA patterns, although the context of organized versus non-organized PA was not considered.

In addition to elucidating distinct patterns of PA among adolescent boys and girls, identification of psychological factors that differ across discrete patterns of PA could prove useful for informing interventions targeting specific subgroups of adolescents. In accordance, prior investigations of latent classes of PA among youth have tended to focus solely on demographic characteristics, which have resulted in a limited understanding of modifiable factors underpinning involvement [14, 15].

Self-determination theory (SDT; [16, 17]) has been widely adopted as a framework for understanding the psychological influences and motivational processes associated with adolescents’ PA. Proponents of SDT posit that satisfaction of psychological needs for competence, relatedness and autonomy foster autonomous motivation, which underpins PA behavior. Higher perceptions of competence, relatedness and autonomy underpin intrinsic motivation to participate in sport and sustain involvement over time [18–20]. Higher perceptions of autonomy support have also been found to promote psychological need satisfaction and intrinsic motivation for organized sport participation [20, 21]. Most of these studies have focused on organized sport without discriminating team and individual sport participation. Thus, it remains unclear whether motivational profiles differ across discrete types of organized and non-organized PA participation.

The current research will attempt to address these shortcomings in the literature by adopting a person-oriented approach to investigate patterns of PA involvement for male and female adolescents using multiple indicators of PA including type, context, duration and intensity. Secondly, PA profiles will be compared on dimensions derived from SDT: intrinsic motivation, competence, relatedness, autonomy and autonomy support, to determine whether psychological processes differ as a function of the pattern of PA undertaken.

## Method

### Participants and procedure

In total, 1004 male and female students took part in the survey. Nine participants did not complete the PA diary in full and were subsequently removed from further analyses. The final sample consisted of 995 students (61.2%; *n* = 609 females, 38.8%; *n* = 386 males) who ranged in age from 12 to 17 years (*M* age = 13.72 years, *SD* = 1.25). Most participants identified as White (71.7%), followed by Black African (7.1%), Asian (5.7%), Mixed (2.1%), and non-identified (13.4%).

The study protocol was approved by the Ethics Committee within the School of Psychology within the university. Sixteen secondary schools located in Dublin, Ireland, were randomly chosen and invited to participate. Eight schools agreed, three granted permission for the researcher to invite students from every year group to participate, while one school granted access to all students in first year. All first and second year students were accessed within two separate schools. The remaining two schools granted access to approximately half of the student classes in Year 3. The participants within this study could therefore be considered representative of the whole school. Researchers distributed information sheets and a parental consent form to adolescents within those schools. The parents of 1891 adolescents were invited to consent to their child’s participation in the research. A total of 1150 (60.8%) parents provided permission, while 45 (3.8%) did not. The remainder did not respond.

Adolescents who returned signed parental consent forms were assembled during a regularly scheduled class and invited to participate. Participants were assured that responses were confidential and would not be shared with school personnel or parents. Willing participants completed an assent form (87.3%), following by a paper-and-pencil survey, which took approximately 40 min to complete.

### Measures

Adolescents completed a physical activity recall seven-day diary (PAR-7DD) and measures of demographic, motivational, social and body image factors. Only data from the PAR-7DD and motivational measures will be utilized in this paper.

#### PAR-7DD

The PAR-7DD was developed for the present study on the basis of the 3-Day Physical Activity Recall (3DPAR), a self-report measure of PA that has previously evidenced sound psychometric properties among young people [22]. Adolescents reported any PA undertaken over the past 7 days and provided details about the type of activity, context of participation (organized/non-organized), duration, and activity intensity (light, moderate, vigorous). Participants were instructed to exclude any time spent in the compulsory physical education class in school. The PAR-7DD differed from the 3DPAR as participants did not have to provide information on activities of daily living or general PA such as chores or part-time work. The recall time frame was also extended. Previous research indicates that adolescents’ PA levels differ on weekday and weekend days, therefore information was sought for the past week to obtain a comprehensive account of young people’s PA patterns [23].

Involvement in extracurricular activities was assessed with one question “Do you play on any sport or PA teams for the school?” Participation in community sports/PA was measured with two questions: “Are you a member of any sport or PA clubs?” and “Do you currently attend any PA classes?” Participants were asked to name the sports/PA in which they engaged.

#### Motivation

The intrinsic motivation subscale of the Behavioral Regulation in Exercise Questionnaire-2 [24] was used to assess motivation towards sport/PA. The subscale contains four items (e.g. “I do PA/sport because it is fun”). Response options ranged from ‘not true for me’ (1) to ‘very true for me’ (5). Items were summed to provide a total score with higher scores indicating higher intrinsic motivation (α = .91).

#### Competence

The Athletic Competence subscale of the Self-Perception Profile for Adolescents [25] was used to measure perceptions of ability in sport/PA. The subscale comprises five items (e.g. ‘Some teenagers feel that they are better than others their age at sports BUT other teenagers don’t feel they can play as well’). Participants selected one response and indicated whether the statement was ‘sort of true’ or ‘really true’ for them. Each item was scored on a scale from 1 to 4 with higher values reflecting greater perceived competence (α = .80).

#### Relatedness

The Acceptance subscale of the Need for Relatedness Scale [26] was used to assess feelings of connection towards other peers within the PA domain. Comprising five items, participants rated the extent to which they feel supported, understood, listened to, valued and safe on a scale ranging from ‘disagree a lot’ (1) to ‘agree a lot’ (5). Item scores were summed to yield a total score with higher scores representing higher perceptions of relatedness (α = .80).

#### Autonomy

The Autonomy subscales of the Basic Needs Satisfaction in Sport Scale [27] comprised ten items that evaluated adolescents’ perceptions of choice, volition and internal perceived locus of causality in PA contexts. Responses options ranged from ‘not true at all’ (1) to ‘very true’ (5). Higher scores reflect increased perceptions of autonomy (α = .89).

#### Autonomy support

The short version of the Sport Climate Questionnaire [28] comprising six items was used to assess the extent to which adolescents perceived their coach/instructor to be autonomy supportive (e.g. “My coach/instructor encourages me to ask questions”). Response options ranged from ‘disagree a lot’ (1) to ‘agree a lot’ (5). Items were summed with higher scores representing increased perceptions of an autonomy-supportive environment (α = .88).

### Statistical analysis

Based on the PAR-7DD, five binary indicators of PA were subsequently derived for each participant. The Compendium of Energy Expenditure for Youth [29], which provides estimated energy costs for different activities at various levels of intensity, was used to assign a metabolic equivalent (MET) to each activity recorded in the PAR-7DD. A MET value of 4 or higher corresponds to activities of at least moderate intensity. Duration of minutes spent in MVPA (i.e. ≥ 4 METs) were calculated separately for each day. Participants were classified as leisure active (1) if they accumulated at least 60 min of leisure time MVPA on three or more days over the past week. If they did not meet these guidelines they were classified as low active (0). The 3 day criterion was selected instead of the recommended guidelines of at least 60 min of MVPA on 7 days because adolescents in this sample were only achieving required amounts of MVPA on a mean of 2.73 (*SD* = 1.67) days. In view of these findings and given that time spent in physical education classes, active travel and general physical activities were excluded from analysis, a lower physical activity threshold was used to dichotomize adolescents as leisure active or low active.

Secondly, respondents were classified as non-organized PA participants (1) if they undertook a minimum of three sessions of non-organized PA over the past week or non-participants (0) if they did not. Non-organized PA was defined as any sport or activity that does not take place alongside a coach or instructor, or constitutes a class, lesson or training session undertaken within an organized school, club or community setting [6]. Any non-organized PA bout that was undertaken for a minimum of 5 min was counted as one session. The 10 most frequent non-organized physical activities reported were soccer (24.6%), walking (20.9%), jogging/running (19.3%), basketball (8.6%), body conditioning exercises (e.g. sit-ups, 6.2%), outdoor play (e.g. tag, skipping, 4.5%), cycling (3.4%), dance (3.0%), swimming (2.5%) and rollerblading (1.5%).

Three binary items were also derived to reflect participation in organized sport/PA: Adolescents who engaged in at least one organized team sport (1) (Gaelic football, 26.6%; soccer, 25.2%; basketball, 17.5%; hockey, 10.0%, hurling, 7.3%; camogie, 6.7%; rugby, 5.7%; volleyball, cricket, baseball, water polo, rowing <1% each); organized individual sport (1) (cross-country running/athletics, 30.8%; swimming, 25.3%; martial arts, 20.8%; badminton, 7.3%; horse-riding, 5.5%; tennis, 4.9%; gymnastics, 2.4%; golf, 1.8%; cycling, handball, sailing <1% each); or organized non-sport PA (1) (dance, 65.2%; gym/exercise class, 34.8%) were classified as participants in each form of activity respectively, whereas those who did not were classified as non-participants (0).

Latent class analysis (LCA) was performed using MPlus 7 [30] to identify distinct homogenous subgroups of adolescents within the dataset based on the five categorical indicators of PA behavior. Conceptual considerations, parsimony and fit criteria were used to decide upon the best fitting model. Models with lower values of Akaike Information Criterion (AIC; [31]), Bayesian Information Criterion (BIC; [32]) and Sample Size Adjusted Bayesian Information Criterion (SSABIC; [33]) indicate improved fit. The Lo-Mendell-Rubin likelihood ratio test ([LMR-LRT; [34]) compares the estimated model with a model with one less class (*k*-1). A non-significant *p*-value indicates that the inclusion of an additional class does not result in a significant improvement in fit. Entropy was used to measure the accuracy with which models classify individuals into their most likely latent class with higher values indicating greater precision [35]. Finally, bivariate residuals are used to evaluate how well a model fits the data, with a lower number of significant residuals indicating superior model fit. Latent class models were run separately for male and female adolescents given the well-established differences in PA levels among this population.

After the optimal number of classes was selected for each gender, one-way Kruskal-Wallis tests were conducted using SPSS version 21 to compare PA profiles on motivational characteristics. The overall significance level was set at *p* < .01. Partial eta squared (η^2^) effect sizes were calculated to interpret the strength of the overall effect with values equal to or greater than .01, .06, and .14, representing small, medium, and large effect sizes, respectively. Mann-Whitney *U* tests followed up significant main effects to determine which PA profile groups differed from one another. To correct for type 1 error owing to multiple comparisons, the significance level was set at .0033 (.05/15) for female and .005 (.05/10) for male comparisons.

## Results

### Latent classes

Two through seven latent classes were estimated separately for male and female adolescents [Table 1 **]**. The model with 6 classes provided the best fit for girls, as indicated by the lower BIC and SSABIC value. The LMR-LRT test indicated that a four-class solution was a better fit than a five-class solution. The four-class model had one significant associated bivariate residual while the six-class model had none, indicating that the four-class model provided a poorer fit to the data. Thus, a six-class solution was selected to represent female PA behavior patterns.

**Table 1:** Model fit statistics of latent class analysis on five physical activity variables

	Number of classes						
		2	3	4	5	6	7
Female							
LL (df)		− 1775.94 (11)	− 1764.61 (17)	− 1759.01 (3)	− 1751.58 (29)	− 1749.54 (5)	− 1748.20 (6)
AIC		3573.88	3563.21	3524.02	3561.15	3509.09	3508.41
BIC		3622.43	3638.24	3537.26	3689.14	3531.15	3534.89
SSABIC		3587.50	3584.27	3527.74	3597.08	3515.28	3515.84
Entropy		0.0	0.80	0.82	0.79	0.82	0.81
No. of sig. residuals		1	1	1	0	0	0
LRT		165.07	22.09	46.61	14.49	123.07	8.45
*p*		.00	.00	.00	.30	.00	.00
Male							
LL (df)		− 1114.78 (11)	− 1102.69 (2)	− 1094.99 (3)	− 1087.43 (4)	− 1085.99 (5)	− 1085.29 (6)
AIC		2251.55	2209.38	2195.97	2182.86	2181.99	2182.59
BIC		2295.07	2217.29	2207.84	2198.69	2201.77	2206.32
SSABIC		2260.17	2210.95	2198.32	2185.99	2185.91	2187.28
Entropy		1.00	0.92	0.94	0.90	0.84	0.90
No. of sig. residuals		2	0	0	0	0	0
LRT		66.03	1760.52	26.58	37.33	455.34	23.92
*p*		.00	.00	.00	.00	.00	.00

*LL(df)* loglikelihood value and associated degrees of freedom, *AIC* Akaike Information Criterion, *BIC* Bayesian information criteria, *SSABIC* sample size adjusted Bayesian information criterion, *LRT* Lo-Mendell-Rubin adjusted likelihood ratio test value

For males, the BIC indicated that a five-class solution most closely approximated the data whilst the SSABIC and AIC pointed towards a six-class model, although values were similar for these classes with less than one unit differentiating the scores. The LMR-LRT did not reach significance. The five-class solution evidenced a higher entropy value in comparison to the six-class model, which coupled with a preference for BIC and parsimony resulted in selection of a five-class model to represent male PA behavior patterns.

Item-response probabilities for each of the observed variables are presented for girls [Table 2] and boys [Table 3] alongside mean minutes spent in daily MVPA to facilitate interpretation of the latent classes. The label ‘leisure active’ was assigned to classes if members performed at least 60 min of leisure time MVPA on three or more days over the past week. Classes that also achieved a minimum of 60 mean minutes of MVPA daily were labelled ‘active’ as they met recommended PA guidelines.

**Table 2:** Response probabilities for each of the physical activity indicators by each latent class for female adolescents

Latent Class	Number	≥60mins /3 days	Non-Organized PA ≥ 3 sessions	Team Sport	Individual Sport	Organized Non-Sport PA	*M* min MVPA/day *M* (*SD*)
1. Organized Run/Swim & Dance/Gym	16	0.00	0.45	0.34	**1.00**	**1.00**	25.89 (8.03)
2. Active Individual Sport	58	**1.00**	0.26	0.39	**1.00**	0.36	70.01 (69.35)
3. Organized Dance	78	0.42	0.21	0.00	0.00	**1.00**	28.31 (24.19)
4. Walk/Run/Outdoor games	104	0.28	**0.78**	0.16	0.09	0.00	23.64 (22.48)
5. Leisure Active Team Sport	140	**0.73**	0.14	**1.00**	0.31	0.37	57.96 (31.93)
6. Non-Participation	213	0.00	0.00	0.09	0.15	0.08	9.85(16.76)

Conditional probabilities >.5 in bold to facilitate interpretation; *MVPA* moderate-to vigorous physical activity, *PA* physical activity

**Table 3:** Response probabilities for each of the physical activity indicators by each latent class for male adolescents

Latent Class	Number	≥60mins /3 days	Non-Organized PA ≥ 3 sessions	Team Sport	Individual Sport	Organized Non-Sport PA	*M* min MVPA/day *M* (*SD*)
1. Leisure Active Gym	11	**0.83**	0.02	0.00	0.00	**1.00**	56.59 (39.91)
2.Leisure Active Individual Sport	68	**0.73**	0.38	0.00	**0.88**	0.06	47.81 (33.79)
3. Non-Participation	89	0.00	0.33	0.32	0.11	0.04	18.42 (17.75)
4. Active Mixed Type	97	**1.00**	**1.00**	**0.72**	0.27	0.09	79.43 (37.53)
5. Active Team Sport	121	**0.91**	0.00	**1.00**	0.34	0.16	71.64 (40.52)

Conditional probabilities >.5 in bold to facilitate interpretation; *MVPA* moderate-to vigorous physical activity, *PA* physical activity

### Female latent classes

Class 1 comprised 2.6% (*n* = 16) of the female sample and reflected high probabilities of engaging in individual sport (cross-country running/athletics, 52.9%; swimming, 29.4%; karate, 5.9%, badminton, 5.9%, horse riding, 5.9%) and organized non-sport PA (dance, 82.4%; exercise classes (e.g. step), 17.6%) but low rates of other PA behaviors. None of the participants in this group achieved 60 min of MVPA on a minimum of 3 days however inspection of raw data indicated that most girls were engaging in recommended levels of MVPA on a maximum of 2 days. This class was labelled ‘*Organized Run/Swim & Dance/Gym*’. Class 2 (*n* = 58, 9.5%) had the highest likelihood of engaging in individual sport (martial arts (e.g. kickboxing, taekwondo, judo, karate, boxing), 23.2%; badminton, 21.8%; cross-country running/athletics, 18.8%; swimming, 14.5%; horse-riding, 11.6%; gymnastics, 4.3%; golf, 2.9%; tennis, 2.9%) and performing at least 60 min MVPA on three or more days with low rates of involvement in other types of PA. In addition, this was the only female class that achieved over 60 mean MVPA minutes daily, achieving recommended PA guidelines. This class was labelled ‘*Active Individual Sport*’. Class 3 (*n* = 78, 12.8%) labelled ‘*Organized Dance*’ was characterized by high rates of participation in organized non-sport PA (dance (e.g. ballet, Irish dance, hip-hop), 94.9%; Gym/exercise classes, 5.1%) and a low likelihood of engaging in other PA behaviors. Class 4 (*n* = 104, 17.1%), named ‘*Walk/Run/Outdoor games*’ demonstrated low levels of participation in all PA apart from non-organized PA, with the most frequent activities comprising walking (42.4%), jogging/running (20.4%), cycling (2.7%), dance (3.4%) informal games (soccer, 7.6%; basketball 2.7%), outdoor play (e.g. tag, skipping, rounders, 7.2%); and body conditioning exercises (e.g. sit-ups, push-ups, 6.0%).

The response pattern discriminated by high rates of MVPA and participation in team sports (Gaelic football, 27.6%; hockey, 22.1%; basketball, 20.3%; camogie/hurling, 16.6%; soccer, 12.4%; volleyball, 0.9%) was identified as Class 5 ‘*Leisure Active Team Sport*’ (*n* = 140, 23%). Class members also averaged roughly 58 mins of MVPA daily, just below recommended PA guidelines. Finally, Class 6, the most prevalent of the female classes (*n* = 213, 35%) comprised girls who had very low probabilities of engaging in the PA behaviors examined and was labelled ‘*Non-Participation*’.

### Male latent classes

Class 1 (*n* = 11, 2.8%) was characterized by high rates of participation in organized non-sport PA and achieving 60 min of MVPA on a minimum of 3 days. Every member of this class attended a gym and lifted weights, used cardiovascular machinery (e.g. treadmill) or participated in fitness classes (e.g. spin) between three and five times a week. Thus, this class was labelled ‘*Leisure Active Gym*’. Class 2 (*n* = 68, 17.6%), was discriminated by high probability of engaging in individual sport (martial arts, 43%; swimming, 25.3%; cross-country running/athletics, 16.5%; tennis, 5.1%; golf, 3.8%; horse-riding, 2.5%; badminton/cycling <2%) and at least 60 min of MVPA on 3 days and was labelled ‘*Leisure Active Individual Sport*’. Class 3 (*n* = 89, 23.1%) was named ‘*Non-Participation*’ as members were characterized by low rates of participation in all PA behaviors.

Representing one quarter (*n* = 97, 25.1%) of adolescent boys, Class 4 was characterized by high rates of participation in a combination of non-organized PA (most frequently reported activities >2%: soccer, 46.3%; jogging/running, 17.3%; basketball, 13.7%; walking, 4.1%; swimming, 3.4%; cycling, 2.7%; hurling, 2.7%; Gaelic football, 2.5%) and organized team sport (soccer, 33.9%; Gaelic football, 27.8%; hurling, 14.8%; basketball, 13.9%; rugby, 7.8%; hockey, baseball <1% each) and high levels of MVPA. As such, this class was labelled ‘*Active Mixed Type*’. Finally, representing the largest grouping, Class 5 (*n* = 121) named ‘*Active Team Sport*’ demonstrated high rates of participation in team sport (soccer, 39.4%; Gaelic football, 25.6%; hurling, 12.8%; rugby, 12.2%; basketball, 8.9%; rowing/water polo <1% each) and high levels of MVPA, and low probabilities of other PA behavior.

### Comparing classes on motivational characteristics

One-way Kruskal-Wallis tests yielded significant effects (*p* < .001) for intrinsic motivation, competence, relatedness, autonomy, and autonomy support among both male [Table 4] and female [Table 5] adolescent PA profiles, with intrinsic motivation demonstrating the largest effect size in each cohort.

**Table 4:** Cross-class comparisons of psychosocial variables across the six physical activity latent classes among female adolescents

Variable	Class 1 Organized Run/Swim & Dance/Gym *n* = 16 *Median (IQR)*	Class 2 Active Individual Sport *n* = 58 *Median (IQR)*	Class 3 Organized Dance *n* = 78 *Median (IQR)*	Class 4 Walk/Run/Outdoor games *n* = 104 *Median (IQR)*	Class 5 Leisure Active Team Sport *n* = 140 *Median (IQR)*	Class 6 Non-Participation *n* = 213 *Median (IQR)*	H	n^2^_p_
Intrinsic Motivation	17.50 (6.0)^a^	19.00 (4.3) b, f	16.00 (6.0) c, g	15.00 (5.8) d, f, h	19.00 (3.8) e, g, h	13.00 (7.0) ^a,^ b, c d, e	112.27**	.18
Competence	12.00 (6.8)	13.5 (6.0) a, b, c	10.50 (5.0) a, d	10.00 (5.0) b, e	14.00 (5.0) d, e, f	10.00 (5.0) c, f	83.84**	.14
Relatedness	22.00 (7.0)	20.00 (8.0)	19.00 (6.0) a	19.00 (9.0) b	22.00 (6.0) a, b, c	18.00 (7.0) c	38.17**	.05
Autonomy	39.50 (11.3)	39.00 (11.0) a	36.00 (11.0)^b, e^	36.50 (12.0)^c, f^	42.00 (9.0)^d, e, f^	32.00 (14.0) a, b, c, d	42.66**	.15
Autonomy Support	21.00 (7.6)	22.00 (8.0) a	22.00 (8.3)	20.5 (8.0) b	24.00 (6.8) b, c	20.00 (8.0) a, c	42.66**	.07

** *p* < .001; Significant pairwise differences between classes are denoted by the same alphabet superscript in each row

**Table 5:** Cross-class comparisons of psychosocial variables across the five physical activity latent classes among male adolescents

Variable	Class 1 Leisure Active Gym *n* = 11 *Median (IQR)*	Class 2 Leisure Active Individual Sport *n* = 68 *Median (IQR)*	Class 3 Non-Participation *n* = 89 *Median (IQR)*	Class 4 Active Mixed Type *n* = 97 *Median (IQR)*	Class 5 Active Team Sport *n* = 121 *Median (IQR)*	H	n^2^_p_
Intrinsic Motivation	15.00 (4.0) a, b	16.00 (6.8) c, d, e	14.00 (5.5) c, f, g	19.70 (2.0) a, d, f	19.00 (3.0) b, e, g	93.87**	.24
Competence	12.00 (4.0) a, b	13.00 (5.8) c, d	11.00 (6.0) e, f	15.00 (4.0) a, c, e	16.18 (4.0) b, d, f	79.97**	.21
Relatedness	18.00 (5.0) a, b	20.00 (7.0) c, d	18.00 (6.0) e, f	23.00 (5.0) a, c, e	22.00 (6.0) b, d, f	51.64**	.11
Autonomy	36.00 (7.0)	39.00 (11.0)a b	34.00 (14.5)c, d	42.00 (7.70)a, c	42.00 (8.0)b, d	45.53**	.13
Autonomy Support	23.00 (3.0)	22.00 (6.8) a, b	18.00 (8.5) c, d	24.00 (8.0) a, c	24.00 (6.0) b, d	57.92**	.15

** *p* < .001; Significant pairwise differences between classes are denoted by the same alphabet superscript in each row

Among the female sample those characterized by a pattern of *Non-Participation* demonstrated significantly lower intrinsic motivation scores (*p* < .0033) than all other latent classes. In turn, members of the *Leisure Active Team Sport* and *Active Individual Sport* profiles reported higher intrinsic motivation than *Walk/Run/Outdoor games* participants (*p* < .002). The *Leisure Active Team Sport* and *Organized Dance* class also differed significantly (*p* < .001), with the former group evidencing higher levels of intrinsic motivation. Regarding perceived competence, girls in the *Leisure Active Team Sport* and *Active Individual Sport* classes demonstrated significantly higher scores compared to those in the *Organized Dance* (*p* < .002), *Walk/Run/Outdoor games* (*p* < .001) and *Non-Participation* (*p* < .001) classes.

In addition, *Leisure Active Team Sport* members significantly differed from girls in the *Organized Dance* (*p* < .001), *Walk/Run/Outdoor games* (*p* = .001) and *Non-Participation* (*p* < .001) classes, demonstrating higher scores on relatedness.

In respect to autonomy, female adolescents from the *Leisure Active Team Sport*, *Active Individual Sport*, *Organized Dance* and *Walk/Run/Outdoor games* classes reported significantly higher (*p* < .001) levels than those characterized by *Non-Participation*. In turn, members of the *Organized Dance* and *Walk/Run/Outdoor games* classes evidenced significantly lower autonomy compared to the girls in the *Leisure Active Team Sport* class (*p* < .001). Finally, for autonomy support, the *Leisure Active Team Sport* and *Active Individual Sport* classes evidenced significantly higher scores (*p* < .001) than the *Non-Participation* class*.* The *Leisure Active Team Sport* and *Walk/Run/Outdoor games* classes also differed significantly (*p* < .001), with the former group demonstrating higher scores.

Among boys, adolescents characterized by a pattern of *Active Team Sport* or *Active Mixed Type* reported significantly higher levels of intrinsic motivation (*p* < .001), competence (*p* < .002) and relatedness (*p* < .005) in comparison to the other latent classes. Additional differences were noted between the *Leisure Active Individual Sport* and *Non-Participation* classes with the latter group demonstrating lower intrinsic motivation (*p* = .003). In relation to perceived autonomy and autonomy support, members of the *Active Team Sport* and *Active Mixed Type* classes evidenced higher levels in comparison to males in the *Leisure Active Individual Sport* and *Non-Participation* classes (*p* < .002). No other significant differences emerged across classes.

## Discussion

The current findings provide a greater understanding of adolescents’ PA patterns and differences in psychological processes associated with PA participation. Previous studies have typically adopted variable-centred approaches (e.g. correlation, factor analysis) that look at relations among isolated variables (e.g. MVPA) based on information about the average person in the sample, which ultimately neglects intra-individual differences [36]. LCA, a person-orientated approach was therefore used in the current study to identify homogeneous subgroups of adolescents that engaged in similar patterns of PA behavior [12].

This study extends previous research [13, 14] providing additional information on the context in which PA is performed and the different ways in which male and female adolescents accumulate MVPA. Overall, boys demonstrated more active patterns of PA participation than girls, which is consistent with prior findings indicating that latent classes characterized by higher levels of MVPA derived from accelerometer and/or self-report measures of PA, comprise a higher proportion of male than female adolescents [15, 37]. In accordance, four of the five male PA profiles were considered leisure active, indicating that recommended levels of MVPA were achieved on at least three days over the past week, with two classes also achieving at least 60 mins of MVPA daily. Thus, boys can accumulate high levels of MVPA through various PA behaviors. Only two of the female classes reflecting discrete patterns of participation in *team sport* and *individual sport*, undertook sufficient MVPA to be considered leisure active, with members of the latter class also engaging in a mean of more than 60 min of MVPA daily.

The findings support prior research in highlighting the significance of organized sport participation in assisting adolescent girls to achieve and sustain higher levels of MVPA [9, 10]. The most prevalent PA profile among girls was *Non-Participation* while for boys it was A*ctive Team Sport* (soccer/Gaelic football/hurling/rugby), with each class representing approximately one third of the respective sample. This result supports previous findings that boys are more likely to engage in moderate-to-vigorous intensity team based activities than girls [38, 39], who are more likely to be characterized by low active patterns [15, 37].

Gender differences were also evident in relation to organized non-sport PA. A small group of male adolescent gym members comprised a leisure active fitness-oriented profile, which contrasted with a lower active pattern of dance among girls. An additional female class *Run/Swim & Dance/Gym* characterized by low levels of MVPA and dual participation in individual sport and organized PA emerged, that was not replicated among the male sample. The results suggest that for girls, partaking in organized dance classes once a week, alone or in addition to a weekly running or swimming training session does not necessarily mean they are classified as being leisure active. Nonetheless, the present findings indicate that organized dance classes can make an important contribution to girls’ overall PA levels with latent class members achieving an average of 25-28 min in MVPA daily. This result is in line with previous research that revealed that female adolescents’ objectively measured activity in structured dance lessons accounted for roughly 29% of their weekly MPVA [11], highlighting the significance of this mode of activity for increasing girls’ PA. Finally, a discrete profile of low active engagement in *non-organized* PA unique to girls emerged that was primarily characterized by participation in individual type activities like walking, jogging and running. This context of PA participation has received limited attention to date, thus this finding makes an important contribution to the existing literature by elucidating that informal exercise reflects a valid and distinct pattern of adolescent girls’ PA, representing 17.1% of the total female sample.

Conversely, for boys dual participation in *non-organized* PA (soccer/run/basketball) alongside *team sport* (soccer, Gaelic football, hurling, basketball) represented a highly active profile of *mixed* PA participation. Of note, roughly half of the non-organized PA bouts reported reflected engagement in informal games of soccer, contrasting girls’ inclination for more individualized types of unstructured activities. Prior research conducted on a nationally representative sample in Ireland revealed that male adolescent sport involvement is dominated by team-based invasion games (i.e. soccer, Gaelic football, basketball, hurling) [40]. This finding contributes to this literature base indicating that preferences for team games are further evident in adolescent boys’ unstructured play. Moreover, the informal nature of non-organized PA, which can be undertaken across multiple settings, alone or with friends, with little cost, commitment or organization, offers a means of accumulating MVPA that is accessible to all young people.

A secondary aim of the study was to determine whether motivational processes derived from SDT differed with respect to the profile of PA undertaken. While previous studies have found that adult sport participants demonstrate higher levels of self-determined motivation and competence than those engaged in fitness activities (e.g. aerobics, weightlifting) [41, 42], this area has received little attention among adolescent samples.

The results revealed that adolescent girls characterized by leisure active participation in organized team or individual sport demonstrated higher levels of intrinsic motivation, competence, relatedness, autonomy and autonomy support relative to those characterized by less active profiles of organized PA (dance/gym), non-organized PA (walk/run) and non-participants.

Consistent with prior research, female adolescents characterized by leisure active sport participation demonstrated significantly higher perceptions of competence than individuals that engaged in less active PA patterns [10, 43]. In accordance, organized sports typically require a certain level of skill to gain entry, with such abilities unnecessary for taking part in unstructured PA (e.g. walking, jogging) or non-competitive pursuits such as dance lessons and gym classes. Qualitative studies further suggest that athletically competent teenagers are more likely to enjoy and pursue competitive sports as it provides a platform to demonstrate their abilities to others [43]. On the basis of these findings it is plausible to suggest that organized PA classes (e.g. spin) or informal exercise may represent a less threatening alternative to competitive sport for girls who lack actual or perceived athletic competence.

Higher perceptions of autonomy and autonomy support also differentiated girls characterized by *leisure active team sport* or *active individual sport* profiles from less active patterns of PA participation. Regular training sessions with coaches targeting improved performance may facilitate increased opportunities for athletes’ input into decision-making regarding skills that necessitate development, which supports feelings of volition over one’s sport participation [44]. Lower levels of autonomy experienced among girls characterized by participation in dance relative to team sport may be a consequence of the class structure in which limited choices are available regarding dance routine adopted. Conversely, the style of instruction adopted may impact female adolescents’ perceived autonomy. Thus, it is unclear whether the structure of the sport or coaching style differentiated participants’ perceptions of autonomy.

Leisure active team sport profiles also demonstrated higher levels of relatedness relative to PA profiles representing *Organized Dance*, *Walk/Run/Outdoor games* and *Non-participants*. This finding may be attributed to the team sport context that comprises individuals working interdependently as part of a group to achieve goals, which ultimately enhances feelings of connection [18, 19, 45].

The findings also revealed that girls characterized by lower active profiles of participation in *Organized Dance*, *Organized Run/Swim & Dance/Gym*, and *Walk/ Run/Outdoor games* demonstrated higher levels of intrinsic motivation and autonomy than non-participants indicating that even low active patterns of leisure PA are associated with more adaptive psychological processes. Prior research indicates that patterns of PA involvement characterized by lower intensity activity (e.g. walking) are more likely to be maintained across adolescence [8]. In addition, personal exercise reflecting swimming, jogging, going to the gym were the only type of activity in which participation was found to increase among Irish teenagers over the secondary school years [40]. These findings underscore the importance of identifying psychological factors associated with low active PA patterns alongside leisure active participation profiles. It should be acknowledged however, that some of the latent female PA classes contained a small number of participants. Further research comprising a bigger sample of adolescent girls characterized by participation in *Organized Dance,* and *Organized Run/Swim & Dance/Gym* is therefore needed for any statistically stronger differences relative to other PA classes to be uncovered.

Furthermore, identifying the motivational characteristics on which girls characterized by low active PA patterns differ from non-participants may also help to identify mechanisms through which inactive individuals can be helped to initiate and engage in a more active lifestyle. The current findings suggest that fostering perceptions of ownership over one’s PA participation, by structuring PA environments to facilitate decision-making opportunities such as offering different PA options within the school or community context may enable girls to identify a form of activity that they enjoy and are motivated to continue [46].

For boys, those characterized by *Active Team Sport* participation alone or in combination with non-organized PA (*Active Mixed Type*) evidenced higher scores on intrinsic motivation, competence, relatedness, autonomy, and autonomy support, relative to boys represented by profiles of *Leisure Active Individual Sport*, *Leisure Active Gym* or *Non-Participation*. However, additional research utilizing a larger sample of male adolescents classified within the *Leisure Active Gym* class in particular, may be necessary to detect any significant differences from other PA groups*.* While, leisure active patterns of organized sport participation irrespective of format undertaken (e.g. team vs individual) was associated with more adaptive psychological profiles relative to less active patterns of PA among girls, this finding was not replicated among boys. In accordance, boys whose profiles included team sport participation differed significantly from their individual sport counterparts, indicating that psychological processes differ dependent on the type of sport (i.e. individual v team) undertaken by males. Prior research exploring predictors of sustained participation in organized sport have typically grouped individuals that performed different types of sport together (e.g. [18]). This has precluded potential differences pertaining to team and individual sport participation to be uncovered.

The current findings suggest that team sport may play a particularly salient role among male youth, perhaps attributable to increased opportunities within the group setting to develop positive relationships that may not be supported within the context of individual activities such an individual sport or gym sessions. Performing as part of a mutually dependent group facilitates multiple opportunities to form connections, foster cooperation and develop positive relationships with teammates, which ultimately contribute to enhanced feelings of relatedness [19, 45]. The greater feelings of belonging, and affiliation arising from high quality relationships with teammates may also account for the higher levels of intrinsic motivation reported among males represented by profiles of team sport participation relative to their individual sport, gym and non-participant counterparts.

In regard to perceived competence, triumph in team sport is dependent on collaborative effort from multiple members whilst individual sport success is contingent on individual performance. Following on from this, losses may be shared among the team or blamed on errors by multiple people whereas failure in individual sport cannot be attributed to anyone but oneself. It is therefore plausible to suggest that the individual sport environment fosters greater one-on-one comparisons than team sport resulting in decreased perceptions of competence. In addition, *leisure active gym* members reported lower levels of competence than those characterized by *active team sport* participation. Boys who lack the necessary skills to gain entry into team sport may opt for this non-competitive fitness alternative that requires little athletic prowess.

Finally, males characterized by patterns of participation in individual sport and team sport significantly differed on perceived autonomy and autonomy support, with the former group performing more poorly. Prior research indicates that participation in individual sports elicits greater pressure than team sport given that performance is contingent on individual as opposed to collective performance [47]. Self-imposed or external pressure from coach or parent to act or think in certain ways undermines feelings of autonomy or sense of ownership over one’s PA participation [19], which may account for the discrepancies evidenced. Further research is necessary to determine whether male adolescents’ autonomy perceptions differ owing to coaching style quality or format of sport undertaken.

The findings offer support for SDT as a framework for exploring motivational and psychological mechanisms underpinning youth PA participation. Most studies to date that have applied SDT to youth PA have typically focused on organized sport or total MVPA, with limited attention directed towards non-organized types of activity such as walking or jogging. The current findings therefore extend the literature by providing a greater insight into the relationship between psychological processes and discrete patterns of organized and non-organized PA participation among adolescents. The results indicate that salience of psychological needs for relatedness, competence and autonomy differs dependent on pattern of PA undertaken. For example, feelings of connection are central to team sport participation but appear to be less important for attending the gym or undertaking informal PA like walking and jogging. Similarly, it appears that perceptions of autonomy may be of greater relevance than perceived competence for maintaining involvement in non-competitive organized PA like dance and fitness classes or more informal games and exercise (e.g. walking).

The cross-sectional nature of the study precludes us however, from making any conclusions regarding the direction of effects between adolescents’ PA patterns and motivational processes or whether latent classes remain stable over time. Longitudinal studies are needed to determine if psychological processes differentially predict sustained involvement, dropout or changing patterns of PA participation during adolescence.

The various profiles of PA participation that emerged have relevance for policies addressing young people’s PA engagement. A consistent finding in this study was that team sport participation was associated with higher levels of MVPA and more adaptive motivational characteristics suggesting that this pattern of activity should be promoted among adolescents. However, adopting such a strategy subsequently neglects individuals who dislike group sports and competitive activities. A simple ‘one size fits all’ approach is not sufficient for promoting participation among teenagers and consequently interventions need to be tailored to reflect different subgroups of adolescents [14]. Whilst many chances exist for young people to take part in sport at an extracurricular or extraschool level, limited opportunities are available for participation in non-sport fitness oriented activities like spinning and aerobics. In addition, non-organized PA such as walking, jogging and cycling could be fostered by increasing the ease with which such activities can be performed, for example, by developing walking and cycling friendly routes that can be safely navigated [10].

The strengths of this study include the relatively large sample size and person-orientated analysis which allowed a more nuanced insight into adolescents’ PA patterns. However, the findings of the study must be considered in view of the study limitations. PA was assessed using a self-report survey. This method has been criticized owing to susceptibility to errors in memory recall, overestimation of PA levels, and socially desirable responses [48]. However, given the relatively large sample size in this study and the need to collect information on the context of PA, the use of costly- and time-intensive objective tools, such as pedometers, was not a viable option [48]. With respect to data analysis, LCA is exploratory in nature and there is no definitive test to facilitate identification of the ‘true’ number of latent classes [49]. However, adolescents’ PA profiles were readily discriminated on motivational characteristics indicating that there were meaningful differences among classes, offering support for the validity of the latent classes [15]. Nevertheless replication of findings among additional samples of secondary school students would offer further support for the validity of these profiles. Finally, class labels were assigned to reflect the defining features of the class but may not encompass the full range of activities undertaken by class members.

## Conclusions

This research offers a novel and nuanced insight into the underlying type of activities that constitute overall patterns of male and female adolescents’ PA and further revealed that psychological processes differ as a function of the pattern of PA undertaken.
